# Supplementary material for: Necrostatin-1 Attenuates Renal Ischemia and Reperfusion Injury via Meditation of HIF-1α/mir-26a/TRPC6/PARP1 Signaling
Source: Mol Ther Nucleic Acids. 2019 Jul 12;17:701–13. doi: 10.1016/j.omtn.2019.06.025 (PMC6706591; doi:10.1016/j.omtn.2019.06.025)
Supplement: Document S1. Figures S1–S5 [file mmc1.pdf]

OMTN, Volume *17*

Supplemental Information

Necrostatin-1 Attenuates Renal Ischemia and Reperfusion Injury via Meditation of HIF-1α/mir-26a/TRPC6/PARP1 Signaling

Bingbing Shen, Mei Mei, Youmin Pu, Huhai Zhang, Hong Liu, Maozhi Tang, Qianguang Pan, Yue He, Xiongfei Wu, and Hongwen Zhao

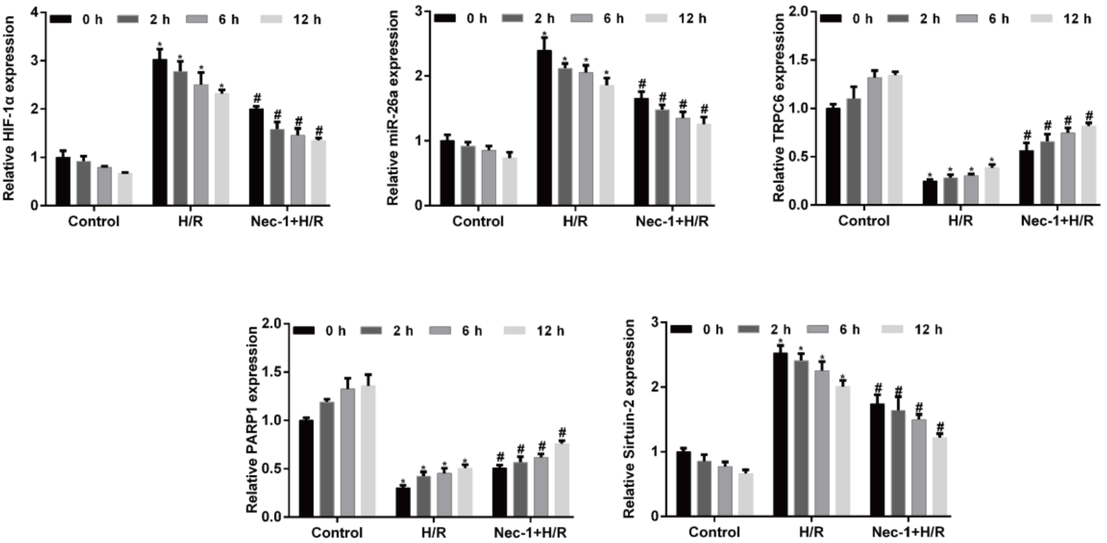

Figure 1S. The levels of HIF-1α, miR-26a, TRPC6, necroptosis, oxidative stress, and inflammation-related molecules in HK-2 cells under conditions of H/R injury with or without Nec-1 treatment. Quantitative real-time PCR was used to determine the relative levels of HIF-1α, miR-26a, TRPC6, PARP1, and Sirtuin-2 in HK-2 cells. *$p < 0.05$ *vs.* control group; #$p < 0.05$ *vs.* the H/R injury group.

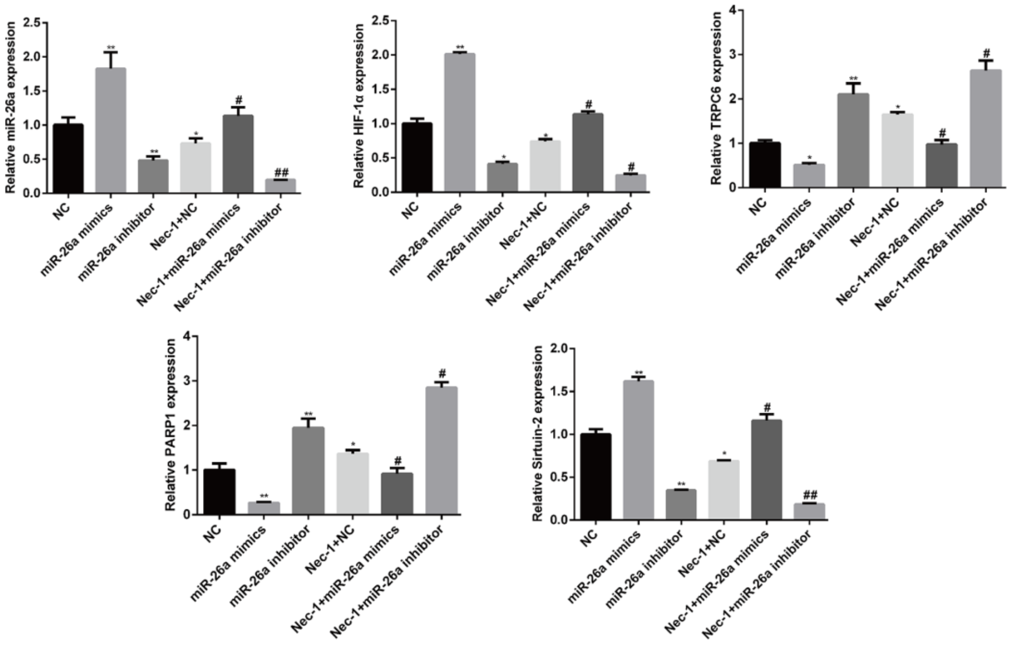

Figure 2S. Effects of downregulation or upregulation of miR-26a on the levels of HIF-1α, miR-26a, TRPC6, necroptosis, oxidative stress, and inflammation-related molecules in HK-2 cells under conditions of H/R injury with or without Nec-1 treatment. Quantitative real-time PCR was used to determine the relative levels of miR-26a, HIF-1α, TRPC6, PARP1, and Sirtuin-2 in HK-2 cells. *$p < 0.05$, **$p < 0.01$ *vs.* the NC group; #$p < 0.05$, ##$p < 0.01$ *vs.* the Nec-1 + H/R group.

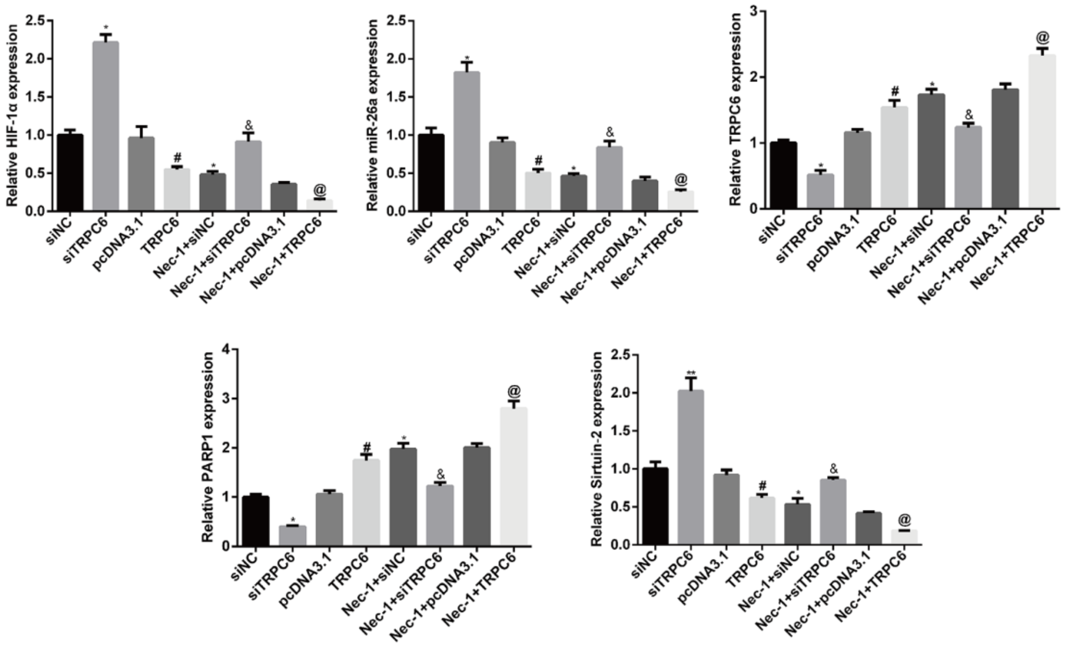

Figure 3S. Effects of TRPC6 overexpression or knockdown on the levels of HIF-1α, miR-26a, TRPC6, necroptosis, oxidative stress and inflammation-related molecules in HK-2 cells under conditions of H/R injury with or without Nec-1 treatment. Quantitative real-time PCR was used to determine the relative levels of miR-26a, HIF-1α, TRPC6, PARP1, and Sirtuin-2 in HK-2 cells. *$p < 0.05$ *vs.* the siNC group; #$p < 0.05$, *vs.* the pcDNA3.1 group; $^{\&}p < 0.05$ *vs.* the Nec-1 + siTRPC6; $^{@}p < 0.05$ *vs.* the Nec-1 + pcDNA3.1 group.

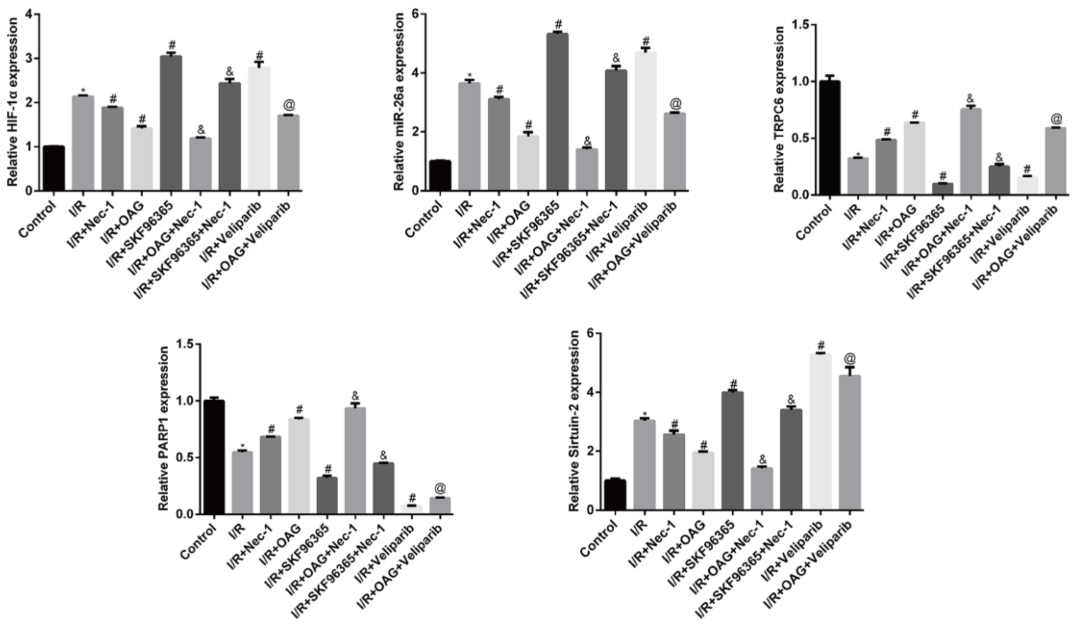

Figure 4S. Nec-1 pretreatment protected rat kidney tissue against I/R-induced oxidative stress and inflammation via the HIF-1α/miR-26a/TRPC6 pathway. Rats in the I/R group were divided into five sub-groups according whether OAG (Sigma, USA), SKF96365 (Sigma, USA) or veliparib (Selleck) was administered before the I/R procedure at 6 h. Similarly, rats in the I/R + Nec-1 group were also divided into three sub-groups. Quantitative real-time PCR was used to determine the relative levels of miR-26a, HIF-1α, TRPC6, PARP1, and Sirtuin-2 in renal tissues. $^{*}p < 0.05$ *vs.* the control group; $\#p < 0.05$ *vs.* the I/R group; $^{\&}p < 0.05$ *vs.* the I/R + Nec-1; $^{@}p < 0.05$ *vs.* the I/R + OAG group.

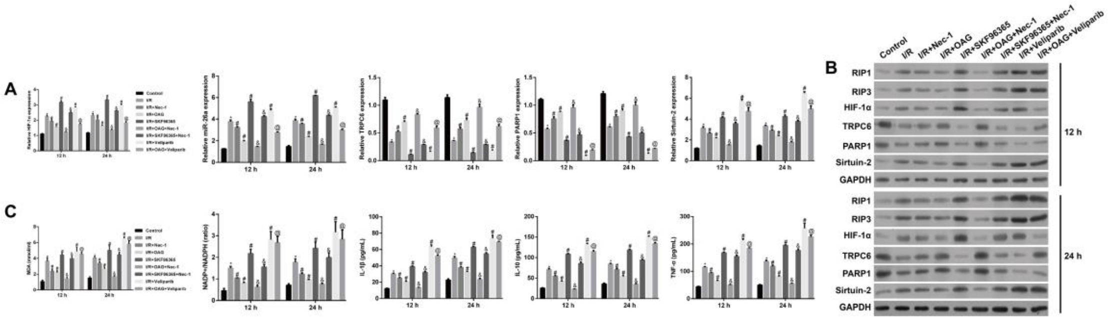

Figure 5S. Nec-1 pretreatment protected rat kidney tissue against I/R-induced oxidative stress and inflammation via the HIF-1α/miR-26a/TRPC6 pathway. Rats in I/R group were divided into five sub-groups according whether OAG (Sigma, USA), SKF96365 (Sigma, USA) or veliparib (Selleck) was administered at before the I/R procedure at 12 and 24 h. Similarly, rats in I/R + Nec-1 group were also divided into three sub-groups. (A) Quantitative real-time PCR was used to determine the relative levels of miR-26a, HIF-1α, TRPC6, PARP1, and Sirtuin-2 in renal tissues. (B) Western blotting was performed to detect HIF-1α, RIP1, RIP3, TRPC6, PARP1, and Sirtuin-2 expression in renal tissues. (C) A MDA detection kit and $NADP^+$/NADPH assay kit were used to measure the MDA level and $NADP^+$/NADPH ratio, respectively. Specific ELISA kits were used to detect the levels of inflammatory cytokines, IL-1β, IL-10, and TNF-α. *$p < 0.05$ *vs.* the control group; #$p < 0.05$ *vs.* the I/R group; $^{\&}p < 0.05$ *vs.* the I/R + Nec-1; $^{@}p < 0.05$ *vs.* the I/R + OAG group.
