# Supplementary material for: Necrostatin-1 Attenuates Renal Ischemia and Reperfusion Injury via Meditation of HIF-1α/mir-26a/TRPC6/PARP1 Signaling
Source: Mol Ther Nucleic Acids. 2019 Jul 12;17:701–13. doi: 10.1016/j.omtn.2019.06.025 (PMC6706591; doi:10.1016/j.omtn.2019.06.025)
Supplement: Document S2. Article plus Supplemental Information [file mmc2.pdf]

Molecular Therapy
Nucleic Acids
Original Article

Necrostatin-1 Attenuates Renal Ischemia and Reperfusion Injury via Meditation of HIF-1α/mir-26a/TRPC6/PARP1 Signaling

Bingbing Shen,[1] Mei Mei,[1] Youmin Pu,[1] Huhai Zhang,[1] Hong Liu,[1] Maozhi Tang,[1] Qianguang Pan,[1] Yue He,[2] Xiongfei Wu,[3] and Hongwen Zhao[1]

[1]Department of Kidney, The First Affiliated Hospital of Army Medical University, Gaotanyan Zhengjie, Shapingba District, Chongqing 400038, China; [2]Department of Urology, General Hospital of Lanzhou, Lanzhou, Gansu, China; [3]Department of Nephrology, Renmin Hospital of Wuhan University (Eastern Hospital), East Lake High-tech Development Zone, Wuhan, Hubei 430200, China

Necroptosis, oxidative stress, and inflammation are major contributors to the pathogenesis of ischemic acute kidney injury. Necrostatin-1 (Nec-1), an inhibitor of the kinase domain of receptor-interacting protein kinase-1 (RIP1), has been reported to regulate renal ischemia and reperfusion (I/R) injury; however, its underlying mechanism of action remains unclear. HK-2 cells were used to create an *in vitro* I/R model, in which the cells were subjected to hypoxia, followed by 2, 6, and 12 h of reoxygenation. For the *in vivo* study, a rat model of renal I/R was established in which samples of rat blood serum and kidney tissue were harvested after reperfusion to assess renal function and detect histological changes. Cell viability and necroptosis were analyzed using the Cell Counting Kit (CCK)-8 assay and flow cytometry, respectively. The expression levels of molecules associated with necroptosis, oxidative stress, and inflammation were determined by real-time PCR, western blotting, immunofluorescence staining, and ELISA. Luciferase and chromatin immunoprecipitation (ChIP) assays were performed to confirm the relevant downstream signaling pathway. We found that pretreatment with Nec-1 significantly decreased hypoxia-inducible factor-1α (HIF-1α) and miR-26a expression, as well as the levels of factors associated with necroptosis (RIP1, RIP3, and Sirtuin-2), oxidative stress (malondialdehyde [MDA], $NADP^+$/NADPH ratio), and inflammation (interleukin [IL]-1β, IL-10, and tumor necrosis factor alpha [TNF-α]) in I/R injury cells and the rat model. However, these effects could be reversed by miR-26a overexpression or TRPC6 knockdown. Mechanistic studies demonstrated that HIF-1α directly binds to the promoter region of *miR-26a*, and that *TRPC6* is a potential target gene for miR-26a. Our findings indicate that Nec-1 can effectively protect against renal I/R injury by inhibiting necroptosis, oxidative stress, and inflammation, and may exert its effects through mediation of the HIF-1α/miR-26a/TRPC6/PARP1 signaling pathway.

INTRODUCTION

Renal ischemia and reperfusion (I/R) injury, the major cause of acute renal failure, typically occurs after renal transplantation or surgery, and is one of the most serious and common health problems seen in the clinic.[1,2] The pathogenic mechanism underlying renal I/R injury is complicated and involves energy metabolism dysfunction,[3] tubular necrosis and apoptosis,[4] inflammation,[5,6] and oxidative stress.[7] The anti-malarial drug, hydroxychloroquine, was shown to markedly reduce the levels of pro-inflammatory cytokines (IL-1β, IL-6, and tumor necrosis factor alpha [TNF-α]) because it exerts its anti-inflammatory effects in the treatment of acute kidney injury.[8] I/R results in excessive reactive oxygen species (ROS) production and antioxidant levels.[9] Administration of a MnSOD mimetic was shown to reduce the severity of a renal I/R injury and increase the expression of antioxidant enzymes.[10] Therefore, gaining a better understanding of the mechanisms of necrosis, inflammation, and oxidative stress that underlie I/R-induced injury would aid in developing new and effective therapies for acute renal failure.

In recent years, there has been increasing interest in the relationship between miRs and I/R injury. For example, several miRs, including miR-21, miR-7, and miR-192, were shown to be upregulated, whereas miR-322 was found to be downregulated in studies of renal I/R models conducted by Godwin et al.[11] and Wei et al.[12] In our previous study, miR-26a, miR-29, and miR-200 were identified as potential novel targets for treating renal I/R injury.[13] Among those possible targets, miR-26a caught our attention because of its participation in renal vascular diseases and interstitial fibrosis.[14] Moreover, our previous bioinformatics study identified transient receptor potential cation channel (*TRPC6*) as an

Received 18 December 2018; accepted 5 June 2019;
https://doi.org/10.1016/j.omtn.2019.06.025.

Correspondence: Xiongfei Wu, Department of Nephrology, Renmin Hospital of Wuhan University (Eastern Hospital), No. 17 High-tech 6th Road, East Lake High-tech Development Zone, Wuhan, Hubei 430200, China.
E-mail: wuxfei@126.com
Correspondence: Hongwen Zhao, Department of Kidney, The First Affiliated Hospital of Army Medical University, No. 30, Gaotanyan Zhengjie, Shapingba District, Chongqing 400038, China.
E-mail: zhaohongwenanu@163.com

This is an open access article under the CC BY license (http://creativecommons.org/licenses/by/4.0/).

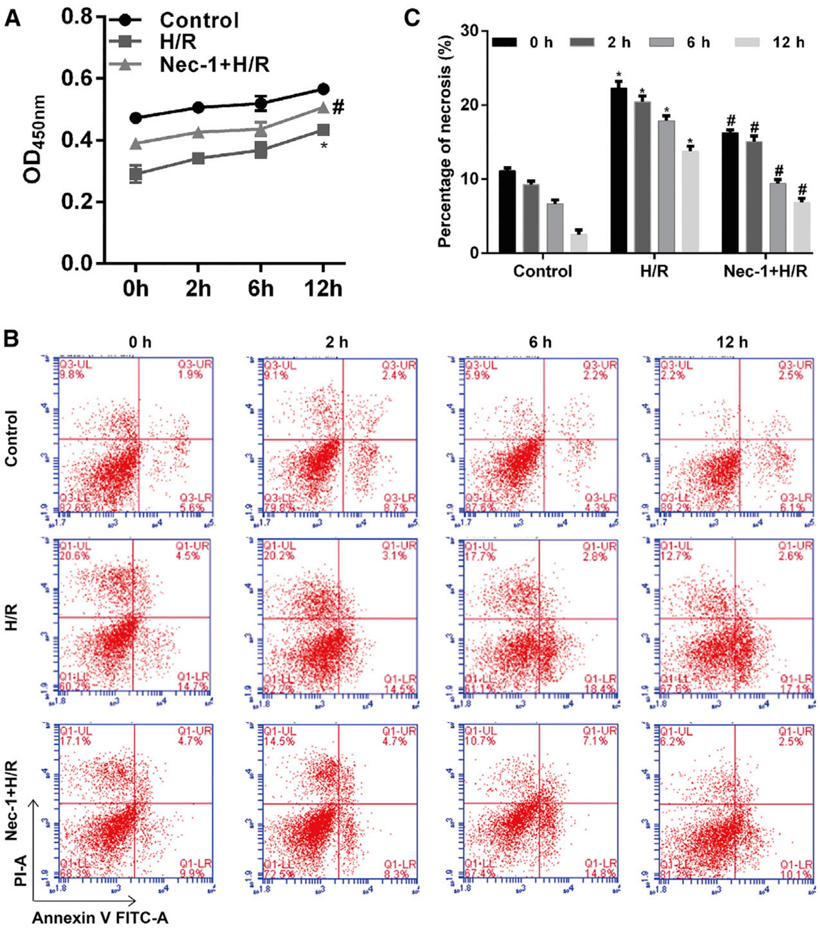

Figure 1. Effects of Nec-1 Treatment on HK-2 Cell Viability and Necrosis under Conditions of H/R Injury

(A) Cell viability was assessed using the CCK-8 assay. (B) Cells were stained with Annexin V-FITC (x axis) and PI (y axis), and analyzed by flow cytometry. (C) Statistical analysis of the percentage of necrotic cells (Annexin V^-/PI^+). *p < 0.05 versus the control group; #p < 0.05 versus the H/R injury group.

upregulated differentially expressed gene involved in the pathogenesis of I/R injury,[13] and also suggested that it could protect against renal I/R injury by inhibiting necroptosis.[15] Furthermore, the TRPC6 activator, OAG, promotes the generation of adenosine diphosphate ribotransferase-1 (PARP-1), which is an enzyme located in the nucleus of eukaryotic cells, and helps to regulate and maintain cellular dynamic balance.[16,17]

Necrostatin-1 (Nec-1), an inhibitor of the kinase domain of receptor-interacting protein kinase-1 (RIP1), was previously reported to protect against renal I/R injury when it is applied before reperfusion.[18] However, the underlying mechanism by which Nec-1 exerts that effect during development of an I/R injury remains unclear. Here, we hypothesized that miR-26a might participate in the protective effect of Nec-1 against I/R injury by regulating signaling pathways associated with necrosis and apoptosis, inflammation, and oxidative stress.

To validate this hypothesis, we created an *in vitro* HK-2 cell model of hypoxia and reoxygenation (H/R) and an *in vivo* rat model of renal I/R injury for use in the present study. We then used these models to explore the underlying mechanism by which Nec-1 regulates a miR-26a mediating downstream pathway in I/R injuries. Our findings provide a novel insight into the anti-necrosis, anti-inflammatory, and anti-oxidative effects of Nec-1 in acute kidney injury.

RESULTS

Nec-1 Treatment Increased Cell Viability and Reduced the Number of Necrotic HK-2 Cells under Conditions of H/R Injury

First, a Cell Counting Kit (CCK)-8 assay was performed to assess the cytotoxicity of Nec-1, which is an inhibitor of the kinase domain of RIP1. As shown in Figure 1A, H/R injury significantly decreased the number of viable cells when compared with control cells ($p < 0.05$). Pretreatment with Nec-1 improved cell viability in a time-dependent manner ($p < 0.05$). Furthermore, a flow cytometric analysis revealed a progressive increase in the number of necrotic cells with increasing reoxygenation time in the H/R injury group; however, that trend was significantly less noticeable among cells pretreated with Nec-1 ($p < 0.05$; Figures 1B and 1C). This suggests that Nec-1 partially protects against H/R-induced necrosis in HK-2 cells.

Nec-1 Treatment Affected the Levels of HIF-1α, miR-26a, TRPC6, Necroptosis, Oxidative Stress, and Inflammation-Related Molecules in HK-2 Cells after H/R Injury

We further detected the levels of hypoxia-inducible factor-1α (HIF-1α) and miR-26a, and found that the increases in HIF-1α (Figure 2A) and miR-26a expression (Figure 1S, $p < 0.05$) induced by H/R injury were inhibited by Nec-1 treatment in a time-dependent manner ($p < 0.05$). The levels of TRPC6 and PARP-1 expression were found to be downregulated following H/R treatment (Figures 2A and S1, $p < 0.05$), and this was consistent with results from our previous study.[15] In contrast, treatment with Nec-1 and H/R increased the levels of TRPC6 and PARP-1 expression, suggesting involvement of the TRPC6/PARP-1 signaling pathway in H/R-induced injuries in HK-2 cells. The levels of RIP1, RIP3, and Sirtuin-2 expression, as key factors in triggering necroptosis, were increased following H/R treatment, and those increases were partially inhibited by Nec-1 (Figures 2A and S1, $p < 0.05$).

In addition, we found an increased accumulation of lipid peroxides with a concordant increase in malondialdehyde (MDA) content in

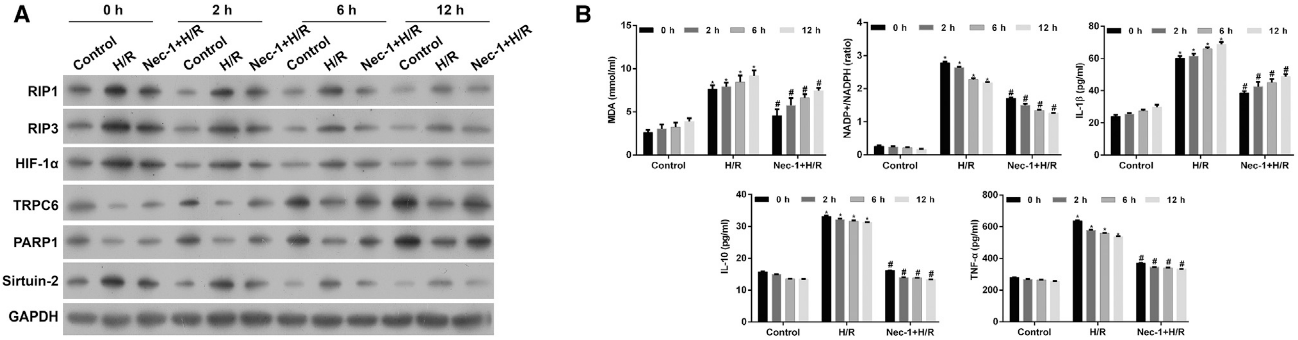

Figure 2. The Levels of HIF-1α, miR-26a, TRPC6, Necroptosis, Oxidative Stress, and Inflammation-Related Molecules in HK-2 Cells under Conditions of H/R Injury with or without Nec-1 Treatment

(A) Western blotting was performed to detect HIF-1α, RIP1, RIP3, TRPC6, PARP1, and Sirtuin-2 expression in HK-2 cells. (B) An MDA detection kit and $NADP^+$/NADPH assay kit were used to measure the MDA level and $NADP^+$/NADPH ratio, respectively. Specific ELISA kits were used to detect the levels of inflammatory cytokines, IL-1β, IL-10, and TNF-α. $^*p < 0.05$ versus control group; $^{\#}p < 0.05$ versus the H/R injury group.

HK-2 cells following H/R treatment; however, those increases were partially reduced by Nec-1 treatment (Figure 2B, $p < 0.05$). We also observed that HK-2 cells in the H/R group showed a significant increase in their intracellular $NADP^+$/NADPH ratios when compared with control cells. The $NADP^+$/NADPH ratios in the Nec-1 + H/R-treated group were significantly lower than those in the solely H/R group (Figure 2B, $p < 0.05$). Moreover, the levels of inflammatory factors IL-1β, IL-10, and TNF-α were also increased in the H/R group (Figure 2B, $p < 0.05$). In contrast, lower levels of IL-1β, IL-10, and TNF-α were found in the Nec-1-treated group when compared with the solely H/R group ($p < 0.05$).

Downregulation of miR-26a Significantly Enhanced the Protective Effects of Nec-1 in HK-2 Cells under Conditions of H/R Injury

Previous studies have shown that miR-26a is involved in renal vascular diseases and interstitial fibrosis.[14] Based on our result showing that miR-26a was overexpressed in H/R injury, we investigated whether miR-26a could have a function in H/R injury with or without Nec-1 treatment. By using the CCK-8 assay (Figure 3A), we found that downregulation of miR-26a significantly increased the viability of HK-2 cells from the H/R group and the Nec-1 + H/R group, whereas this effect was reversed by upregulation of miR-26a. Consistent with that finding, the percentage of cells undergoing necrosis in response to H/R with or without Nec-1 treatment was significantly decreased by miR-26a downregulation, but increased by miR-26a overexpression (Figures 3B and 3C, $p < 0.05$).

At the molecular level, downregulation of miR-26a significantly reduced miR-26a, HIF-1α, Sirtuin-2, RIP1, and RIP3 expression, but increased TRPC6 and PARP1 expression, whereas upregulation of miR-26a gave the opposite results as determined by quantitative real-time PCR (Figure S2, $p < 0.05$, $p < 0.01$), western blotting (Figure 4A), and immunofluorescence staining (Figure 4B), respectively. Additionally, downregulation of miR-26a decreased and upregulation of miR-26a increased the cellular content of MDA, the intracellular $NADP^+$/NADPH ratio, and the levels of inflammatory factors IL-1β, IL-10, and TNF-α in HK-2 cells in response to H/R, and these effects were even more apparent after Nec-1 treatment (Figure 4C, $p < 0.05$).

TRPC6 Overexpression Significantly Enhanced the Protective Effects of Nec-1 in HK-2 Cells after H/R Injury

We investigated the effects of TRPC6 on the protective effects of Nec-1 in HK-2 cells after H/R injury. CCK-8 assays showed that *TRPC6* knockdown significantly suppressed cell viability in the H/R group and the Nec-1 + H/R group. However, cell viability in both of those groups was significantly increased after induction of TRPC6 overexpression (Figure 5A, $p < 0.05$).

Moreover, TRPC6 overexpression decreased, whereas TRPC6 knockdown increased the percentage of cells undergoing necrosis in response to H/R with or without Nec-1 treatment, as determined by flow cytometry assays (Figures 5B and 5C, $p < 0.05$). In agreement with the effects produced by downregulation of miR-26a, TRPC6 overexpression significantly reduced miR-26a, HIF-1α, Sirtuin-2, RIP1, and RIP3 expression but increased TRPC6 and PARP1 expression in HK-2 cells in response to H/R, as determined by quantitative real-time PCR (Figure S3, $p < 0.05$), western blotting (Figure 6A), and immunofluorescence staining (Figure 6B), respectively. We also examined the effects of TRPC6 overexpression or knockdown on oxidative stress and inflammation-related molecules in HK-2 cells. As shown in Figure 6C, TRPC6 overexpression reduced and TRPC6 knockdown increased the cellular content of MDA, the intracellular $NADP^+$/NADPH ratio, and the levels of IL-1β, IL-10, and TNF-α in HK-2 cells in response to H/R with or without Nec-1 treatment ($p < 0.05$).

miR-26a, Mediated by HIF-1α, Directly Targeted TRPC6 in HK-2 Cells in Response to H/R

HIF-1α expression was significantly increased in HK-2 cells in response to H/R. Furthermore, miR-26a expression was clearly

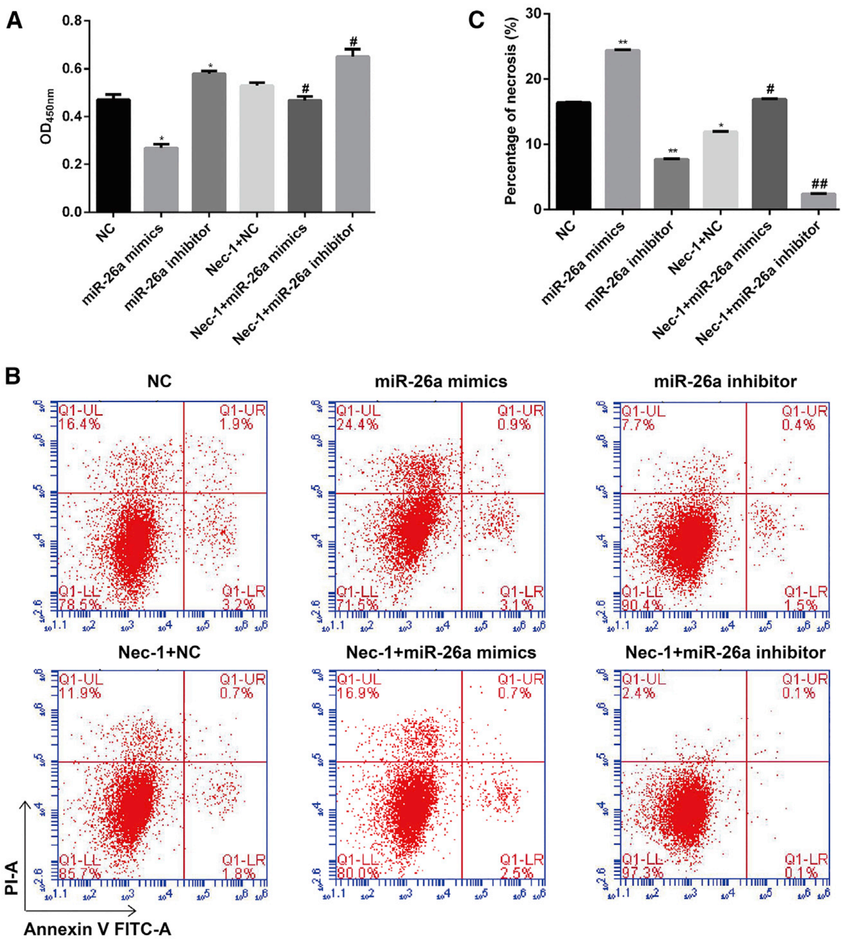

Figure 3. Effects of Downregulation or Upregulation of miR-26a on the Viability and Necrosis of HK-2 Cells under Conditions of H/R Injury with or without Nec-1 Treatment

(A) Cell viability was assessed using the CCK-8 assay. (B) Cells were stained with Annexin V-FITC (x axis) and PI (y axis), and analyzed by flow cytometry. (C) Statistical analysis of the percentage of necrotic cells (Annexin V^-/PI^+). *$p < 0.05$, **$p < 0.01$ versus the NC group; $^{\#}p < 0.05$, $^{\#\#}p < 0.01$ versus the Nec-1 + H/R group.

upregulated. These results suggested that HIF-1α is a critical factor that affects *miR-26a* transcription. Therefore, we established a luciferase reporter gene plasmid carrying a sequence upstream of the *miR-26a* promoter region, and co-transfected it along with pcDNA-HIF-1α into the cells. As shown in Figure 7A, the luciferase reporter assay demonstrated that pcDNA-HIF-1α transfection significantly increased the luciferase activity of miR-26a-WT (wild-type) cells when compared with that of control cells ($p < 0.001$), whereas there was no significant change in the luciferase activity of miR-26a-MUT (mutant) cells. A chromatin immunoprecipitation (ChIP) analysis confirmed that direct binding of HIF-1α to the promoter regions of *miR-26a* and *HIF-1α* could promote the transcription of *miR-26a* in HK-2 cells (Figures 7B and 7C, $p < 0.01$).

Our previous study suggested that TRPC6 might protect against renal I/R injury by inhibiting necroptosis via increasing PARP1 levels.[15] Given the remarkably reduced levels of necrosis in HK-2 cells upon H/R after downregulation of miR-26a or TRPC6 overexpression, we examined whether *TRPC6* might be a target gene of miR-26a. Examinations of the TargetScan, PicTar, and miRanda databases allowed us to identify the potential binding sequence in the TRPC6 3′ UTR; this sequence is shown in Figure 7D. Subsequently, a luciferase reporter assay showed that co-transfection of the TRPC6-3′ UTR-WT and miR-26a mimics led to a significant decrease in luciferase activity (Figure 7E, $p < 0.001$). These results demonstrated that HIF-1α/miR-26a/TRPC6 signaling was involved in the protective role of Nec-1 in HK-2 cells under conditions of H/R injury.

Nec-1 Pretreatment Protected Rat Kidney Tissue against I/R-Induced Oxidative Stress and Inflammation via the HIF-1α/miR-26a/TRPC6 Pathway

To further investigate the protective role of Nec-1 in renal I/R injury, we established an *in vivo* rat model of renal I/R in which groups of rats were subjected to either sham surgery or I/R surgery (with or without Nec-1 pretreatment). As shown in Figures S4 and 8A, the increases in HIF-1α, miR-26a, RIP1, RIP3, and Sirtuin-2 levels in renal tissues were inhibited by Nec-1, and the decreases in TRPC6 and PARP1 were reversed by Nec-1. Moreover, OAG treatment markedly reversed the I/R-induced increases in HIF-1α, miR-26a, RIP1, RIP3, and Sirtuin-2 expression, and reductions in TRPC6 and PARP1 levels. Administration of SKF96365 or veliparib further enhanced cell necrosis in the I/R and I/R + Nec-1 groups after reoxygenation for 6, 12, or 24 h, respectively ($p < 0.05$). Furthermore, the levels of MDA, the intracellular $NADP^+$/NADPH ratios, and the levels of inflammatory factors IL-1β, IL-10, and TNF-α in blood serum were all increased by I/R and were significantly higher than those in the sham control group ($p < 0.05$). Administration of Nec-1 significantly reduced the levels of MDA, the intracellular $NADP^+$/NADPH ratios, and the levels of inflammatory factors IL-1β, IL-10, and TNF-α ($p < 0.05$). Similarly, OAG treatment imitated the effects of Nec-1, whereas the opposite effects were observed when either SKF96365 or veliparib was used (Figure 8B; $p < 0.05$). Here, we present only the data gathered at 6 h; data for the other time points (12 and 24 h) are presented in Figure S5.

Effect of Nec-1 Pretreatment on Renal Histopathological Changes and Immunohistochemical Analysis Results in a Rat Model of Renal I/R

As shown in Figure 9A, cytoplasmic vacuoles, tubular dilatation, cytoplasmic spaces, and tubular cell necrosis were all observed in the I/R group after reoxygenation for 12 h, but were absent in the sham control group. However, there were fewer histological alterations in the I/R + Nec-1, I/R + OAG, and I/R + OAG + Nec-1 groups, with the fewest being found in the I/R + OAG + Nec-1 group. Notably, histological alterations were significantly increased in tissue specimens from the I/R + SKF96365, I/R + SKF96365 + Nec-1, I/R + veliparib, and I/R + OAG + veliparib groups when compared with the I/R group, and the most alterations were found in the I/R + veliparib group.

An immunohistochemical analysis showed increases in RIP1 and RIP3 expression, and decreases in TRPC6 and PARP1 expression in renal tissues induced by I/R, as compared with the renal tissues from the sham control group. Administration of Nec-1 with or without OAG significantly reduced RIP1 and RIP3 levels, but increased TRPC6 and PARP1 levels in renal tissues, whereas application of SKF96365 or veliparib increased the expression of necroptosis-related proteins (RIP1, RIP3, TRPC6, and PARP1) induced by I/R (Figure 9B).

DISCUSSION

In this study, we found that administration of Nec-1 protected against renal I/R injuries prior to reperfusion by suppressing necrosis, inflammation, and oxidative stress, which is in agreement with the findings of Linkermann et al.[19] Mechanistically, Nec-1 pretreatment significantly downregulated the levels of HIF-1α, miR-26a, RIP1, RIP3, Sirtuin-2, and MDA; reduced intracellular $NADP^+$/NADPH ratios; and also reduced the levels of inflammatory factors IL-1β, IL-10, and TNF-α in an HK-2 cell model of H/R and a rat model of renal I/R injury.

It is well accepted that hypoxia is crucial for a renal I/R injury, in which tissues and cells produce a series of hypoxic responses that involve complex molecular mechanisms.[20] HIF-1α release is an important adaptive response of cells to hypoxia, and HIF-1α degradation is inhibited under hypoxic conditions.[21] Other studies showed that HIF binding sites are present in most hypoxia-related *miRNA* (HRM) promoters, suggesting that HIF may be the key regulator of HRM expression under hypoxic conditions.[22] Here, we found that HIF-1α could promote *miR-26a* expression by directly binding to its promoter region, indicating a significant role for HIF-α in the upregulation of miR-26a in I/R injury. Interestingly, miR-26a also positively regulated HIF-α expression in a HK-2 cell model of H/R injury, which suggests that HIF-1α and miR-26a regulation may be interdependent. Similar results were also reported regarding the association between miR-21 and HIF-α.[20] Our previous study *in vitro* study showed that TRPC6 could protect against renal I/R injury, possibly by inhibiting necroptosis.[15] However, our findings were opposite of those reported by Li et al.,[23] who showed that inhibition of TRPC6 enhanced the levels of α-ketoglutarate and promoted hydroxylation of HIF-1α to suppress HIF-1α accumulation in glioma cells, which might be explained by different diseases and cell types.

In this study, *TRPC6* was confirmed as a potential target gene for miR-26a. We further manipulated miR-26a expression and investigated its regulatory effects on *TRPC6* in HK-2 cells. *TRPC6* was validated as a direct target of miR-26a and was suppressed by miR-26a overexpression. These results indicated that miR-26a not only acts with HIF-α to form a positive feedback loop, but also directly inhibits TRPC6 activity in our HK-2 cell model of H/R injury.

We also observed that miR-26a overexpression or TRPC6 knockdown caused an increase in RIP1, RIP3, Sirtuin-2, and MDA levels, $NADP^+$/NADPH ratios, and the levels of IL-1β, IL-10, and TNF-α, whereas knockdown of miR-26a or TRPC6 overexpression attenuated these changes, indicating that the use of an anti-miR-26a agent could be a strategy for preventing renal necroptosis. Related studies reported that renal cells undergo apoptosis, necrosis, or necroptosis following I/R.[24,25] The execution of necroptosis requires the assembly of a RIP1 and/or RIP3-containing necroptosome.[26–28] Consistent with our results, RIP1 was previously shown to contribute to renal I/R injury, which could be alleviated by Nec-1 pretreatment.[18] Furthermore, RIP1 has been reported to be deacetylated in a sirtuin-2-dependent manner and be associated with PARP-1 activation.[29,30] Therefore, we speculated that as an inhibitor of the kinase domain of RIP1, Nec-1 might exert its protective effect in I/R injuries via the miR-26a-targeted TRPC6 regulation of necroptosis-related proteins. MDA is a lipid peroxidation product that decreases activity of the ADP- and NADH-dependent mitochondrial respiratory chains, and thereby causes disturbances in membranous Na^+K^+ ATPase activity.[31] Oxidative stress increases the cellular $NADP^+$/NADPH ratio, which is associated with antioxidative mechanisms.[32] In addition, Yasuda et al.[33] reported that the levels of inflammatory cytokines IL-10 and TNF-α were reduced in chloroquine, inhibiting sepsis-induced acute kidney injuries.

In summary, our results provide the first evidence that Nec-1 pretreatment exerts renoprotective effects in I/R injuries, and identified a previously unknown pathway comprising HIF-1α, miR-26a, TRPC6, and PARP1 that regulates renal necroptosis, oxidative stress, and inflammation. However, it is undeniable that the effects of Nec-1 on I/R injury may be mediated by a variety of mechanisms in addition to the HIF-1α/miR-26a/TRPC6/PARP1 pathway, and further investigations are required to uncover any other mechanisms involved.

MATERIALS AND METHODS

HK-2 Cell Culture and H/R Injury Induction

All animal experiments were approved by the Institutional Animal Care and Use Committee of the First Affiliated Hospital of Army Medical University (Congqing, China). Human renal tubular epithelial HK-2 cells were obtained from the Kunming Cell Bank of the Chinese Academy of Sciences (Kunming, China). The cells were cultured in DMEM/F12 medium (Hyclone, Logan, UT, USA) supplemented with 10% fetal bovine serum (FBS; GIBCO, Grand Island, NY, USA)

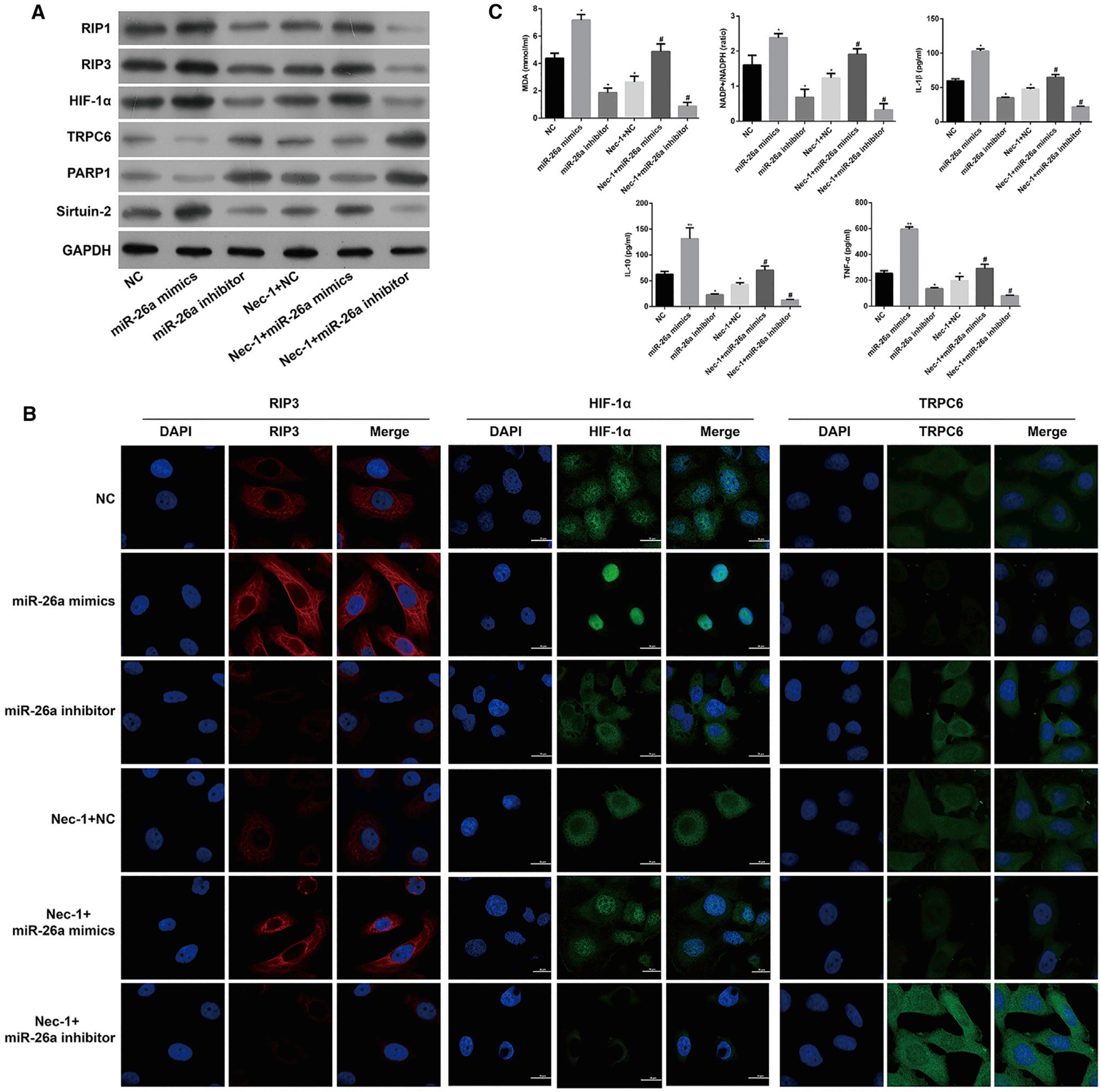

Figure 4. Effects of Downregulation or Upregulation of miR-26a on the Levels of HIF-1α, miR-26a, and TRPC6 Expression, Necroptosis, Oxidative Stress, and Inflammation-Related Molecules in HK-2 Cells under Conditions of H/R Injury with or without Nec-1 Treatment

(A) Western blotting was performed to detect HIF-1α, RIP1, RIP3, TRPC6, PARP1, and Sirtuin-2 expression in HK-2 cells. (B) Immunofluorescence images showing RIP3, HIF-1α, and TRPC6 expression, respectively. (C) An MDA detection kit and $NADP^+$/NADPH assay kit were used to measure the MDA level and $NADP^+$/NADPH ratio, respectively. Specific ELISA kits were used to detect the levels of inflammatory cytokines, IL-1β, IL-10, and TNF-α. *$p < 0.05$, **$p < 0.01$ versus the NC group; $^{\#}p < 0.05$, $^{\#\#}p < 0.01$ versus the Nec-1 + H/R group.

in a 37°C humidified incubator with an atmosphere of 5% CO_2. The cells were randomly divided into the following three groups: (1) a control group, in which the cells were incubated under normoxic conditions; (2) a H/R group, in which the cells were exposed to 4–6 h of hypoxia followed by 4–6 h of reoxygenation (95% air and 5% CO_2); and (3) a Nec-1 + H/R group, in which after 4–6 h of hypoxia, Nec-1 (30 mmol/L) was added to the medium, followed by 4–6 h of reoxygenation. Subsequently, the three groups were exposed to hypoxia

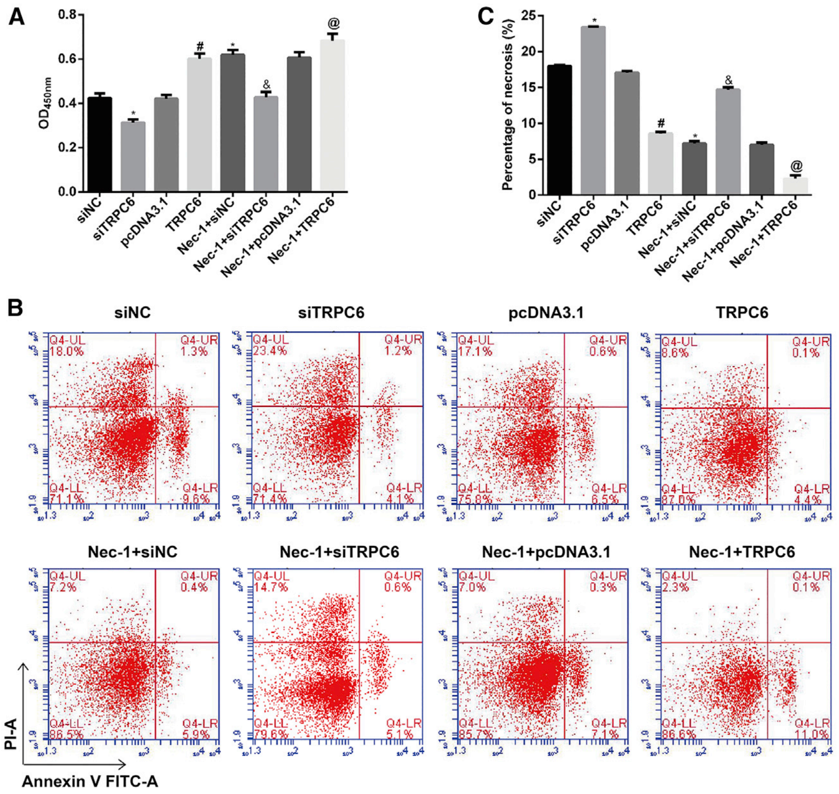

Figure 5. Effects of TRPC6 Overexpression or Knockdown on the Viability and Necrosis of HK-2 Cells under Conditions of H/R Injury with or without Nec-1 Treatment

(A) Cell viability was assessed using the CCK-8 assay. (B) Cells were stained with Annexin V-FITC (x axis) and PI (y axis), and analyzed by flow cytometry. (C) Statistical analysis of the percentage of necrotic cells (Annexin V^-/PI^+). $^*p < 0.05$, compared with the siNC group; $^{\#}p < 0.05$ versus the pcDNA3.1 group; $^{\&}p < 0.05$ versus the Nec-1 + siTRPC6 group; $^{@}p < 0.05$ versus the Nec-1 + pcDNA3.1 group.

(5% CO_2, 1% O_2, and 94% N_2) for 6 h followed by reoxygenation (5% CO_2, 21% O_2, and 74% N_2) for 0, 2, 6, and 12 h, respectively.

Cell Groups and Transfection

The miR-26-a mimics, miR-26-a inhibitor, and negative control (NC) were synthesized by RiBoBio (Guangzhou, China). The pcDNA3.1-TRPC6 (TRPC6), pcDNA3.1-control vector, siTRPC6, and small interfering RNA targeting negative control (siNC) were purchased from Santa Cruz Biotechnology (Dallas, TX, USA). All cell transfections were performed using Lipofectamine 2000 (Invitrogen, Carlsbad, CA, USA). After 48 h of transfection, the cells were harvested and used for *in vitro* experiments.

Animal Models of Renal I/R Injury

Male Sprague-Dawley rats (weight, 180–220 g) were purchased from the Slac Laboratory Animal Center (Shanghai Slac Laboratory Animal, China) and randomly assigned to three groups: (1) a sham-operated control group (n = 9), (2) an I/R group (n = 45), and (3) an I/R + Nec-1 group (n = 27). After ~7 days of acclimatization and 12 h of fasting, the rat model of renal I/R injury was established. In brief, the rats were fasted overnight and then anesthetized with 1% pentobarbital sodium (50 mg/kg; Sigma-Aldrich, St. Louis, MO, USA) administered by intraperitoneal injection. Each anesthetized rat was then placed on a heating pad to maintain a constant body temperature. Subsequently, rats underwent a 45-min occlusion of the left renal pedicle, followed by 24 h of reperfusion. Rats in the sham group were subjected to the same procedure but without any vessel occlusion. Rats in the I/R + Nec-1 group received 2 mL Nec-1 by intragastric administration (2 mg/kg; Hangzhou Moshadong Pharmaceutical, Hangzhou, China) 30 min prior to occlusion.

Intervention Effects of OAG, SKF96365, or Veliparib during I/R Injury

A TRPC6 inhibitor or PARP1 inhibitor was administered during I/R injury. Rats in the I/R group were divided into five groups (n = 3 rats per group) according to whether OAG (Sigma, USA), SKF96365 (Sigma, USA), or veliparib (Selleck) was administered before the I/R procedure. Similarly, rats in the I/R + Nec-1 group were also divided into three groups (n = 3 rats per group). The rats were sacrificed at different time points (6, 12, or 24 h after reperfusion), and their kidneys were harvested. Next, sections of kidney tissue were fixed in 10% buffered formalin for histological examination. The remaining kidney tissue from each rat was frozen in liquid nitrogen and stored at −80°C for further analysis.

Cell Proliferation Assay

The CCK-8 assay was used to evaluate the proliferation of HK-2 cells in the different groups. In brief, HK-2 cells were treated with CCK-8 (10 μL/well; Sigma, USA) at the end of the indicated time point (0, 2, 6, and 12 h, respectively) and then incubated for 2 h. The absorbance at 450 nm was recorded using a microplate reader (Bio-Rad, Hercules, CA, USA).

Flow Cytometry

HK-2 cells were harvested, resuspended in PBS, and then successively stained with 5 μL of Annexin V-fluorescein isothiocyanate (FITC; Beyotime Biotechnology, Shanghai, China) for 5 min, followed by staining with 10 μL of propidium iodide (PI; Beyotime Biotechnology, Shanghai, China) for 15 min in the dark. The stained cells were analyzed by flow cytometry (FACSCalibur; BD Biosciences, Franklin Lakes, NJ, USA).

Quantitative Real-Time PCR

Total RNA was extracted from experimental HK-2 cells or the rat kidney tissues by using an RNA Purified Total RNA Extraction

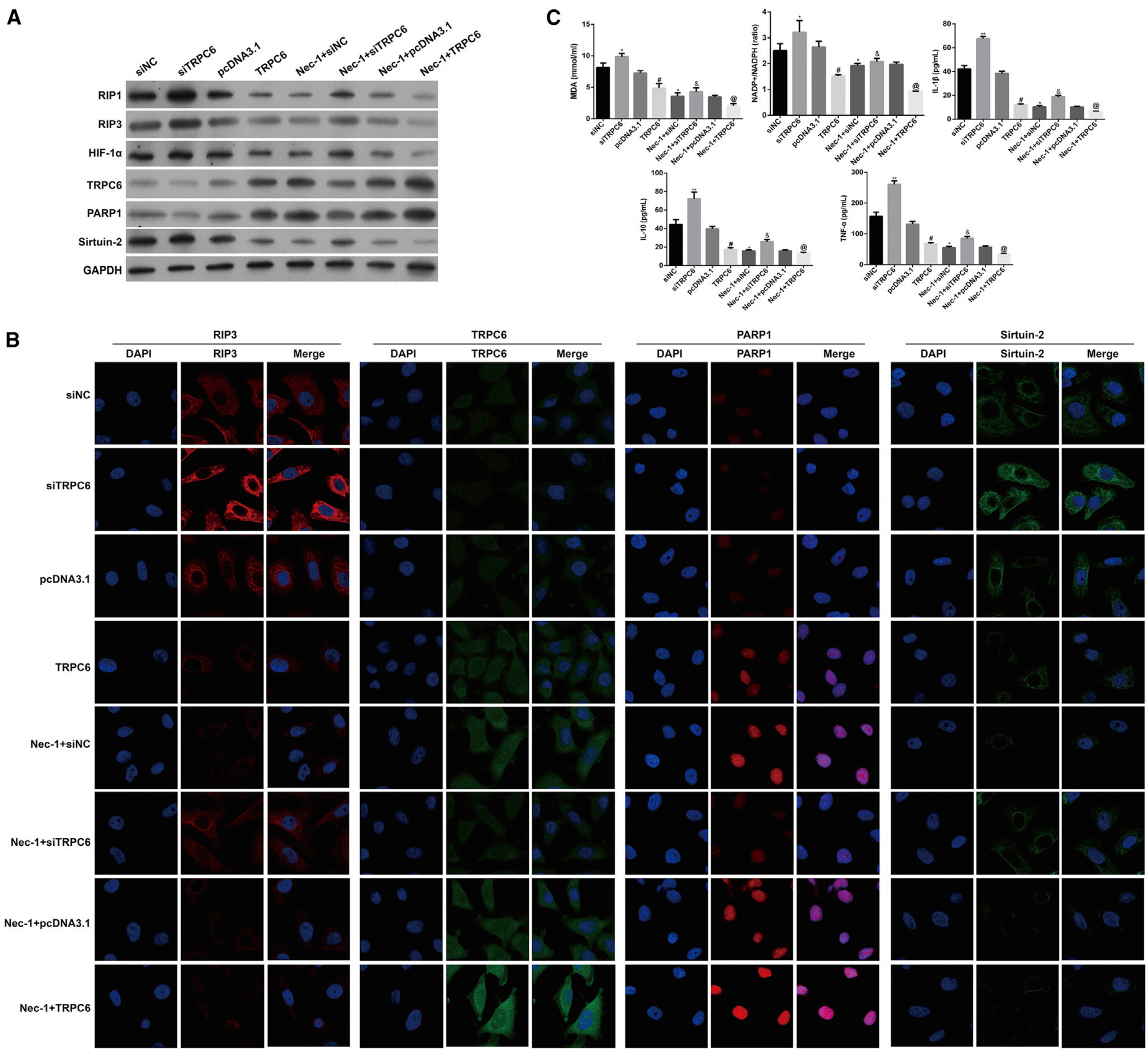

Figure 6. Effects of TRPC6 Overexpression or Knockdown on the Levels HIF-1α, miR-26a, TRPC6, Necroptosis, Oxidative Stress, and Inflammation-Related Molecules in HK-2 Cells under Conditions of H/R Injury with or without Nec-1 Treatment

(A) Western blotting was performed to detect HIF-1α, RIP1, RIP3, TRPC6, PARP1, and Sirtuin-2 expression in HK-2 cells. (B) Immunofluorescence images showing RIP3, TRPC6, PARP1, and Sirtuin-2 expression, respectively. (C) An MDA detection kit and $NADP^+$/NADPH assay kit were used to measure the MDA level and $NADP^+$/NADPH ratio, respectively. Specific ELISA kits were used to detect the levels of inflammatory cytokines, IL-1β, IL-10, and TNF-α. $^*p < 0.05$ versus the siNC group; $^\#p < 0.05$ versus the pcDNA3.1 group; $^\&p < 0.05$ versus the Nec-1 + siTRPC6 group; $^@p < 0.05$ versus the Nec-1 + pcDNA3.1 group.

Kit (BioTeke, Beijing, China) according to the manufacturer's instructions. Real-time PCR was performed with a 7900 HT Real-Time PCR System (Applied Biosystems, Foster City, CA, USA). The primers used for RT-PCR were synthesized by Sangon Biotech (Shanghai, China) and are listed in Table 1. The relative levels of expression of the targeted molecules were calculated by an Exicycler 96 PCR System (Bioneer, Alameda, CA, USA) that used the $2-\Delta\Delta Ct$ method. The expression levels were normalized to that of U6 or GAPDH, which served as internal reference genes.

Western Blotting

Western blotting was performed using primary antibodies against RIP1 (1:1,000, 17519-1-AP; Proteintech, Rosemont, IL,

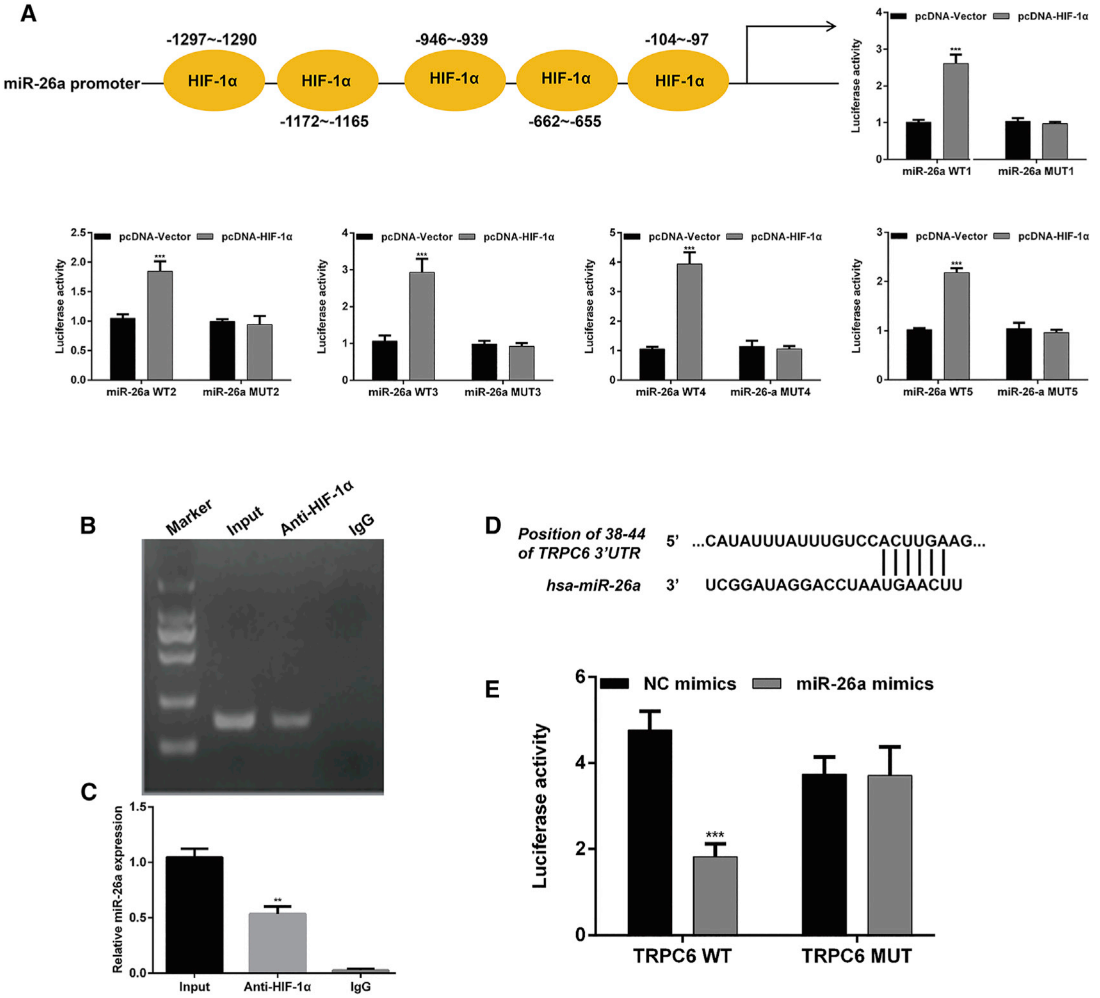

Figure 7. HIF-1α/miR-26a/TRPC6 Signaling Was Involved in the Protective Role of Nec-1 in HK-2 Cells under Conditions of H/R Injury

(A) The five regions of HIF-1α that bound with miR-26a are shown. *MiR-26a* was identified as a target gene of HIF-1α in HK-2 cells. Five wild-type (WT) or the corresponding six mutant miR-26a 3′ UTRs were co-transfected with pcDNA-HIF-1α or pcDNA-Vector into HK-2 cells; luciferase activity was measured by the dual-luciferase reporter assay. ***$p < 0.001$, compared with the pcDNA-Vector; ***$p < 0.001$, compared with the pcDNA-Vector. (B) ChIP assays revealed that HIF-1α directly bound to the promoter region of *miR-26a* and enhanced the promoter's activity. (C) Real-time PCR analysis of miR-26a expression. **$p < 0.001$, compared with input. (D) Sequences of the TRPC6 3′ UTR and miR-26a as predicted by bioinformatics databases (Targetscan, PicTar, and miRanda). (E) Luciferase activity was measured by the dual-luciferase reporter assay. ***$p < 0.001$ versus NC mimics.

USA), RIP3 (1:1,000, 17563-1-AP; Proteintech), HIF-1α (1:1,000, PB0245; BOSTER Biological Technology, Pleasanton, CA, USA), TRPC6 (1:400, BA3394; BOSTER Biological Technology), PARP1 (1:1,000, PB0343; BOSTER Biological Technology), Sirtuin-2 (1:1,000; PB0174; BOSTER Biological Technology), and GAPDH (1:10,000, RC-5G5; KangChen Bio-tech, Shanghai, China), followed by incubation with a horseradish peroxidase-conjugated goat anti-rabbit immunoglobulin G (IgG) antibody (1:20,000, BA1054; BOSTER Biological Technology) for 1 h at room temperature. The signals were detected by enhanced chemiluminescence (Pierce, Rockford, IL, USA).

Detection of MDA Levels

MDA levels were detected to evaluate oxidative stress by using an MDA detection kit (A003-1; Nanjing Jiancheng Bioengineering Company, China) according to the manufacturer's instructions. Enzyme activity is presented in units of mmol/mL.

Measurement of $NADP^+$/NADPH Concentrations and Ratios

The concentrations of NADP and NADPH were measured by using an $NADP^+$/NADPH assay kit (ECNP-100) (BioAssay Systems, Hayward, CA, USA) according to the manufacturer's instructions. Standards included with the kits were used to prepare calibration curves, and $NADP^+$/NADPH ratios were

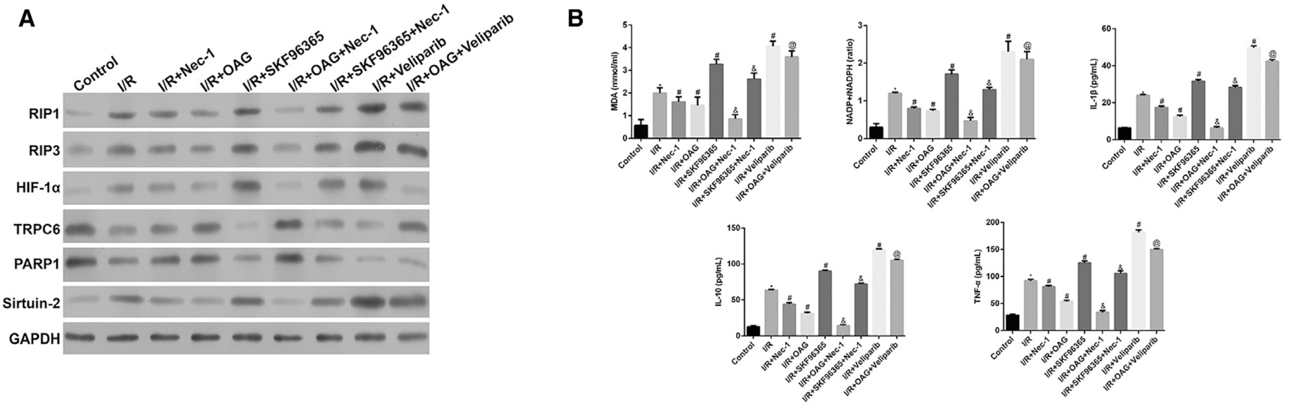

Figure 8. Nec-1 Pretreatment Protected Rat Kidney Tissue against I/R-Induced Oxidative Stress and Inflammation via the HIF-1α/miR-26a/TRPC6 Pathway

Rats in the I/R group were divided into five sub-groups according to whether OAG (Sigma, USA), SKF96365 (Sigma, USA), or veliparib (Selleck) was administered before the I/R procedure at 6 h. Similarly, rats in the I/R + Nec-1 group were also divided into three sub-groups. (A) Western blotting was performed to detect HIF-1α, RIP1, RIP3, TRPC6, PARP1, and Sirtuin-2 expression in renal tissues. (B) An MDA detection kit and $NADP^+$/NADPH assay kit were used to measure the MDA level and $NADP^+$/NADPH ratio, respectively. Specific ELISA kits were used to detect the levels of inflammatory cytokines, IL-1β, IL-10, and TNF-α. $^*p < 0.05$ versus the control group; $^{\#}p < 0.05$ versus the I/R group; $^{\&}p < 0.05$ versus the I/R + Nec-1 group; $^{@}p < 0.05$ versus the I/R + OAG group.

calculated by measuring the intensity of the reduced product color at 460 nm.

Measurement of Inflammatory Cytokines

The levels of interleukin-1β (IL-1β), interleukin-10 (IL-10), and TNF-α in cells or blood serum were measured by using specific ELISA kits (BOSTER Biological Technology, USA) according to the manufacturer's guidelines (EK0392, EK0416, and EK0525, respectively).

Immunofluorescence Staining

For immunofluorescence staining, cultured HK-2 cells were fixed with 4% buffered paraformaldehyde in PBS and then washed with PBS for 5 min. Next, the cells were incubated overnight at 4°C with primary antibodies against RIP3 (17563-1-AP; Proteintech), HIF-1α (PB0245; BOSTER Biological Technology), TRPC6 (BA3394; BOSTER Biological Technology), PARP1 (PB0343; BOSTER Biological Technology), and Sirtuin-2 (PB0174; BOSTER Biological Technology), followed by incubation with a FITC-labeled anti-goat secondary antibody (Beyotime Institute of Biotechnology). The cell nuclei were stained with DAPI (Beyotime, Shanghai, China) for 20 min at room temperature. Immunofluorescence was examined under an inverted fluorescence microscope (Olympus Corporation, Japan).

Dual-Luciferase Reporter Assay

The full-length human HIF-1α code sequence was sub-cloned into a pcDNA3.1 vector and served as the pcDNA3.1-HIF-1α plasmid. The WT and MUT sequences of the pri-miR-26a promoter were designed by GeneCopoeia (Rockville, MD, USA). The 3′ UTR of TRPC6 containing miR-26a binding sites and its corresponding MUT 3′ UTR were also synthesized by GeneCopoeia. These 3′ UTRs were cloned into a pGL3-promoter vector (Promega, Madison, WI, USA) to generate WT pGL3-miR-26a, MUT pGL3-miR-26a, WT pGL3-TRPC6-3′ UTR, and MUT pGL3-TRPC6-3′ UTR, respectively. Next, 293T cells were seeded in a 96-well plate and cultured overnight. For HIF-1α and miR-26a, 293T cells were transfected with pcDNA-vector or pcDNA-HIF-1α, followed by co-transfection with a Renilla luciferase vector (Promega, USA) plus 200 ng of the pGL3 reporters containing WT pGL3-miR-26a or MUT pGL3-miR-26. For miR-26a and TRPC6, 293T cells were transfected with NC mimics or miR-26a mimics, followed by co-transfection with Renilla luciferase vector (Promega, USA) plus 200 ng of the pGL3 reporters containing the WT pGL3-TRPC6-3′ UTR or MUT pGL3-TRPC6-3′ UTR. After 48 h of transfection, luciferase activity was determined using the dual-luciferase reporter assay system (Promega, USA).

ChIP Assay

ChIP assays were performed according to instructions provided with a SimpleCHIP Enzymatic ChIP Kit (Cell Signaling Technology, Danvers, MA, USA). In brief, cells were harvested with SDS buffer, pelleted by centrifugation, and re-suspended in immunoprecipitation (IP) buffer. The cross-linked chromatin samples were immunoprecipitated overnight with HIF-1α antibody. Nonspecific IgG was used as a technical control. Immunoprecipitated DNA was analyzed by qPCR, and the specific primers used for the miR-26a promoter were as follows: forward: 5′-CTCAACTGGTGTCGTGGAGTCGG CAATTCAGTTGAGAGCCTATC-3′ and reverse: 5′-ACACTCCAG CTGGGTTCAAGTAATCCAGGATA-3′. Images were quantified using ImageJ analysis software.

Histological Evaluation

In brief, the kidney tissues were embedded in paraffin and cut into 4-μm sections. After mounting on glass slides, the sections were deparaffinized with xylene, dehydrated with ethanol, and then stained

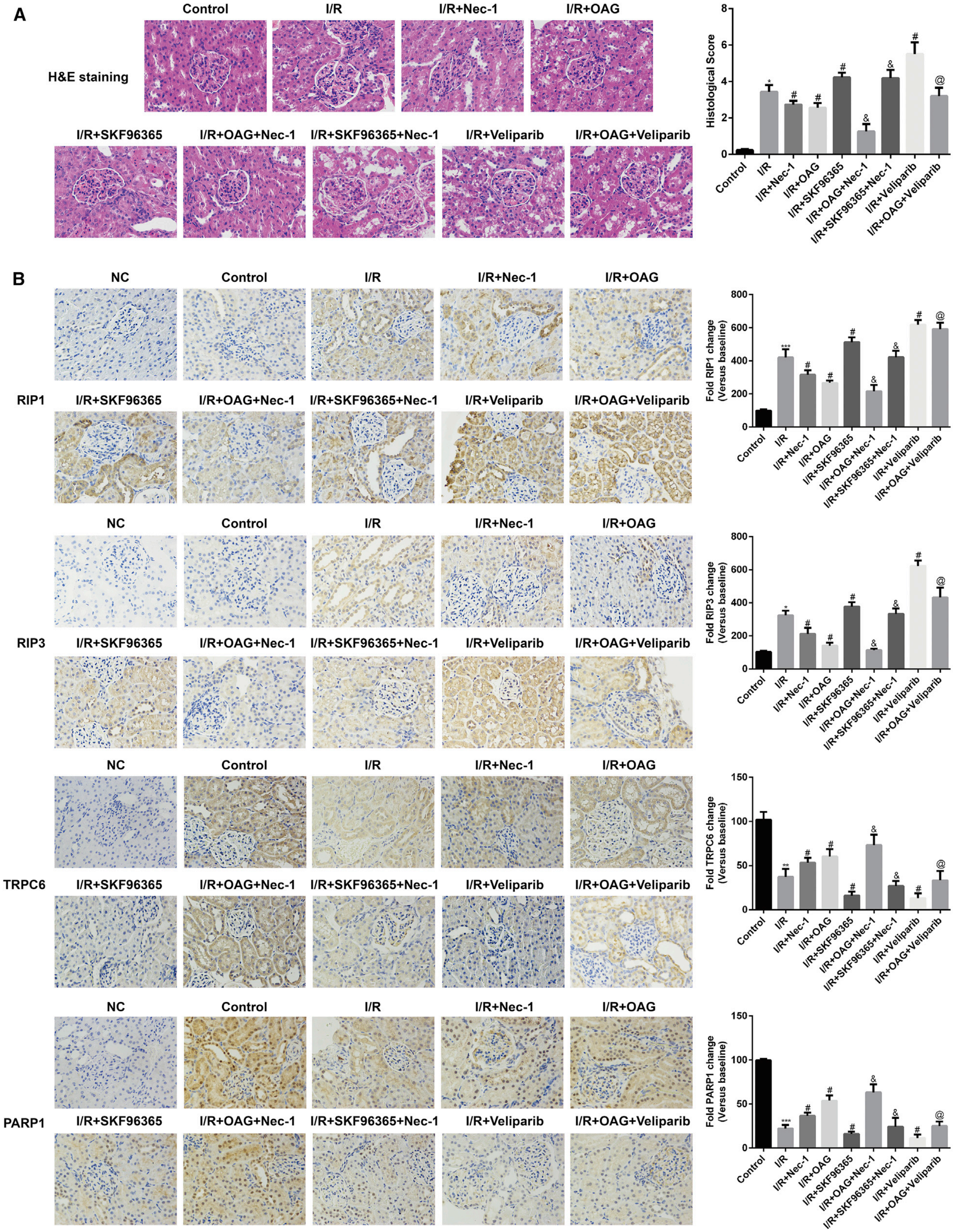

(legend on next page)

Table 1. Primers for Quantitative Real-Time PCR

Gene	Forward (5′–3′)	Reverse (5′–3′)
miR-26a	CTCAACTGGTGTCGTGGAGTCGGCAATTC AGTTGAGAGCCTATC	ACACTCCAGCTGGGTTCAAGTAAT CCAGGATA
HIF-1α	GAACGTCGAAAAGAAAAGTCTCG	CCTTATCAAGATGCGAACTCACA
U6	CTCGCTTCGGCAGCACA	AACGCTTCACGAATTTGCGT
TRPC6	GTGATCGCTCCACAAGCCTAT	CTGCCAACTGTAGGGCATTCT
PARP1	CGGAGTCTTCGGATAAGCTCT	TTTCCATCAAACATGGGCGAC
Sirtuin-2	TGCGGAACTTATTCTCCCAGA	GAGAGCGAAAGTCGGGGAT
GADPH	TGTTCGTCATGGGTGTGAAC	ATGGCATGGACTGTGGTCAT

with H&E. The stained sections were examined under an optical microscope at ×200 magnification for the following parameters: hemorrhage, cytoplasmic vacuole formation, tubular dilatation, and tubular cell necrosis.

Immunohistochemistry (IHC)

Paraffin-embedded sections of kidney tissue were prepared (5-μm thickness) and incubated in antigen retrieval buffer (citrate [pH 6]) for 40 min at 95°C. Next, the sections were immersed in 3% H_2O_2 for 15 min in the dark, washed with PBS, and then incubated overnight at 4°C with primary antibodies against RIP1 (17519-1-AP; Proteintech), RIP3 (17563-1-AP; Proteintech), TRPC6 (BA3394; BOSTER Biological Technology), and PARP1 (PB0343; BOSTER Biological Technology). After being rinsed three times in PBS, the sections used for immunoperoxidase labeling were incubated for 1 h at room temperature with a horseradish peroxidase-conjugated secondary antibody. After rinsing, all the stained slides were viewed under an Olympus BX50 microscope (Olympus Corporation, Japan).

Statistical Analysis

Results for continuous variables are presented as the mean ± SD. Significant differences were determined using GraphPad 6.0 software (USA). ANOVA and post hoc Bonferroni analysis were performed for multiple comparisons. The p values <0.05 were considered to be statistically significant.

SUPPLEMENTAL INFORMATION

Supplemental Information can be found online at https://doi.org/10.1016/j.omtn.2019.06.025.

AUTHOR CONTRIBUTIONS

B.S., M.M., Y.P., X.W., and H.Z. designed and performed the experiments. B.S., H.Z., H.L., M.T., and Q.P. analyzed and interpreted the data. B.S., M.M., Y.H., X.W., and H.Z. wrote the paper.

CONFLICTS OF INTEREST

The authors declare no competing interests.

ACKNOWLEDGMENTS

This work was supported by the National Natural Science Foundation of China (grant 81570610).

REFERENCES

1. Lee, D., Park, S., Bae, S., Jeong, D., Park, M., Kang, C., Yoo, W., Samad, M.A., Ke, Q., Khang, G., and Kang, P.M. (2015). Hydrogen peroxide-activatable antioxidant prodrug as a targeted therapeutic agent for ischemia-reperfusion injury. Sci. Rep. *5*, 16592.
2. Malek, M., and Nematbakhsh, M. (2015). Renal ischemia/reperfusion injury; from pathophysiology to treatment. J. Renal Inj. Prev. *4*, 20–27.
3. Xu, Y.M., Ding, G.H., Huang, J., and Xiong, Y. (2016). Tanshinone IIA pretreatment attenuates ischemia/reperfusion-induced renal injury. Exp. Ther. Med. *12*, 2741–2746.
4. Toronyi, E. (2008). [Role of apoptosis in the kidney after reperfusion]. Orv. Hetil. *149*, 305–315.
5. Thurman, J.M. (2007). Triggers of inflammation after renal ischemia/reperfusion. Clin. Immunol. *123*, 7–13.
6. Ornellas, F.M., Ornellas, D.S., Martini, S.V., Castiglione, R.C., Ventura, G.M., Rocco, P.R., Gutfilen, B., de Souza, S.A., Takiya, C.M., and Morales, M.M. (2017). Bone marrow-derived mononuclear cell therapy accelerates renal ischemia-reperfusion injury recovery by modulating inflammatory, antioxidant and apoptotic related molecules. Cell. Physiol. Biochem. *41*, 1736–1752.
7. Wang, W., Jittikanont, S., Falk, S.A., Li, P., Feng, L., Gengaro, P.E., Poole, B.D., Bowler, R.P., Day, B.J., Crapo, J.D., and Schrier, R.W. (2003). Interaction among nitric oxide, reactive oxygen species, and antioxidants during endotoxemia-related acute renal failure. Am. J. Physiol. Renal Physiol. *284*, F532–F537.
8. Tang, T.T., Lv, L.L., Pan, M.M., Wen, Y., Wang, B., Li, Z.L., Wu, M., Wang, F.M., Crowley, S.D., and Liu, B.C. (2018). Hydroxychloroquine attenuates renal ischemia/reperfusion injury by inhibiting cathepsin mediated NLRP3 inflammasome activation. Cell Death Dis. *9*, 351.
9. Aragno, M., Cutrin, J.C., Mastrocola, R., Perrelli, M.G., Restivo, F., Poli, G., Danni, O., and Boccuzzi, G. (2003). Oxidative stress and kidney dysfunction due to ischemia/reperfusion in rat: attenuation by dehydroepiandrosterone. Kidney Int. *64*, 836–843.

Figure 9. Effect of Nec-1 Pretreatment on Renal Histopathological Changes and Immunohistochemical Analysis Results in a Rat Model of Renal I/R

Rats in the I/R group were divided into five sub-groups according to whether OAG (Sigma, USA), SKF96365 (Sigma, USA), or veliparib (Selleck) was administered before the I/R procedure. Similarly, rats in I/R + Nec-1 group were also divided into three sub-groups. (A) Sections of kidney tissue were stained with H&E and subjected to histological examination to detect renal tubule injuries. (B) Immunohistochemical analysis of RIP1, RIP3, TRPC6, and PARP1 in renal tissues. *p < 0.05 versus the control group; #p < 0.05 versus the I/R group; &p < 0.05 versus the I/R + Nec-1 group; @p < 0.05 versus the I/R + OAG group.

10. Kim, J., Kil, I.S., Seok, Y.M., Yang, E.S., Kim, D.K., Lim, D.G., Park, J.W., Bonventre, J.V., and Park, K.M. (2006). Orchiectomy attenuates post-ischemic oxidative stress and ischemia/reperfusion injury in mice. A role for manganese superoxide dismutase. J. Biol. Chem. *281*, 20349–20356.

11. Godwin, J.G., Ge, X., Stephan, K., Jurisch, A., Tullius, S.G., and Iacomini, J. (2010). Identification of a microRNA signature of renal ischemia reperfusion injury. Proc. Natl. Acad. Sci. USA *107*, 14339–14344.

12. Wei, Q., Bhatt, K., He, H.Z., Mi, Q.S., Haase, V.H., and Dong, Z. (2010). Targeted deletion of Dicer from proximal tubules protects against renal ischemia-reperfusion injury. J. Am. Soc. Nephrol. *21*, 756–761.

13. Shen, B., Zhou, S., He, Y., Zhao, H., Mei, M., and Wu, X. (2013). Revealing the underlying mechanism of ischemia reperfusion injury using bioinformatics approach. Kidney Blood Press. Res. *38*, 99–108.

14. Zhu, X.Y., Ebrahimi, B., Eirin, A., Woollard, J.R., Tang, H., Jordan, K.L., Ofori, M., Saad, A., Herrmann, S.M., Dietz, A.B., et al. (2015). Renal Vein Levels of MicroRNA-26a Are Lower in the Poststenotic Kidney. J. Am. Soc. Nephrol. *26*, 1378–1388.

15. Shen, B., He, Y., Zhou, S., Zhao, H., Mei, M., and Wu, X. (2016). TRPC6 May Protect Renal Ischemia-Reperfusion Injury Through Inhibiting Necroptosis of Renal Tubular Epithelial Cells. Med. Sci. Monit. *22*, 633–641.

16. Wang, Y., Dawson, V.L., and Dawson, T.M. (2009). Poly(ADP-ribose) signals to mitochondrial AIF: a key event in parthanatos. Exp. Neurol. *218*, 193–202.

17. Nagayama, T., Simon, R.P., Chen, D., Henshall, D.C., Pei, W., Stetler, R.A., and Chen, J. (2000). Activation of poly(ADP-ribose) polymerase in the rat hippocampus may contribute to cellular recovery following sublethal transient global ischemia. J. Neurochem. *74*, 1636–1645.

18. Linkermann, A., Bräsen, J.H., Himmerkus, N., Liu, S., Huber, T.B., Kunzendorf, U., and Krautwald, S. (2012). Rip1 (receptor-interacting protein kinase 1) mediates necroptosis and contributes to renal ischemia/reperfusion injury. Kidney Int. *81*, 751–761.

19. Linkermann, A., Heller, J.O., Prókai, A., Weinberg, J.M., De Zen, F., Himmerkus, N., Szabó, A.J., Bräsen, J.H., Kunzendorf, U., and Krautwald, S. (2013). The RIP1-kinase inhibitor necrostatin-1 prevents osmotic nephrosis and contrast-induced AKI in mice. J. Am. Soc. Nephrol. *24*, 1545–1557.

20. Xu, X., Kriegel, A.J., Jiao, X., Liu, H., Bai, X., Olson, J., Liang, M., and Ding, X. (2014). miR-21 in ischemia/reperfusion injury: a double-edged sword? Physiol. Genomics *46*, 789–797.

21. Yee Koh, M., Spivak-Kroizman, T.R., and Powis, G. (2008). HIF-1 regulation: not so easy come, easy go. Trends Biochem. Sci. *33*, 526–534.

22. Fasanaro, P., D'Alessandra, Y., Di Stefano, V., Melchionna, R., Romani, S., Pompilio, G., Capogrossi, M.C., and Martelli, F. (2008). MicroRNA-210 modulates endothelial cell response to hypoxia and inhibits the receptor tyrosine kinase ligand Ephrin-A3. J. Biol. Chem. *283*, 15878–15883.

23. Li, S., Wang, J., Wei, Y., Liu, Y., Ding, X., Dong, B., Xu, Y., and Wang, Y. (2015). Crucial role of TRPC6 in maintaining the stability of HIF-1α in glioma cells under hypoxia. J. Cell Sci. *128*, 3317–3329.

24. Havasi, A., and Borkan, S.C. (2011). Apoptosis and acute kidney injury. Kidney Int. *80*, 29–40.

25. Linkermann, A., De Zen, F., Weinberg, J., Kunzendorf, U., and Krautwald, S. (2012). Programmed necrosis in acute kidney injury. Nephrol. Dial. Transplant. *27*, 3412–3419.

26. Declercq, W., Vanden Berghe, T., and Vandenabeele, P. (2009). RIP kinases at the crossroads of cell death and survival. Cell *138*, 229–232.

27. Vandenabeele, P., Galluzzi, L., Vanden Berghe, T., and Kroemer, G. (2010). Molecular mechanisms of necroptosis: an ordered cellular explosion. Nat. Rev. Mol. Cell Biol. *11*, 700–714.

28. Christofferson, D.E., and Yuan, J. (2010). Necroptosis as an alternative form of programmed cell death. Curr. Opin. Cell Biol. *22*, 263–268.

29. Xu, X., Chua, C.C., Kong, J., Kostrzewa, R.M., Kumaraguru, U., Hamdy, R.C., and Chua, B.H. (2007). Necrostatin-1 protects against glutamate-induced glutathione depletion and caspase-independent cell death in HT-22 cells. J. Neurochem. *103*, 2004–2014.

30. Jouan-Lanhouet, S., Arshad, M.I., Piquet-Pellorce, C., Martin-Chouly, C., Le Moigne-Muller, G., Van Herreweghe, F., Takahashi, N., Sergent, O., Lagadic-Gossmann, D., Vandenabeele, P., et al. (2012). TRAIL induces necroptosis involving RIPK1/RIPK3-dependent PARP-1 activation. Cell Death Differ. *19*, 2003–2014.

31. Humphries, K.M., Yoo, Y., and Szweda, L.I. (1998). Inhibition of NADH-linked mitochondrial respiration by 4-hydroxy-2-nonenal. Biochemistry *37*, 552–557.

32. Korolczuk, A., Caban, K., Amarowicz, M., Czechowska, G., and Irla-Miduch, J. (2016). Oxidative Stress and Liver Morphology in Experimental Cyclosporine A-Induced Hepatotoxicity. BioMed Res. Int. *2016*, 5823271.

33. Yasuda, H., Leelahavanichkul, A., Tsunoda, S., Dear, J.W., Takahashi, Y., Ito, S., Hu, X., Zhou, H., Doi, K., Childs, R., et al. (2008). Chloroquine and inhibition of Toll-like receptor 9 protect from sepsis-induced acute kidney injury. Am. J. Physiol. Renal Physiol. *294*, F1050–F1058.

OMTN, Volume *17*

Supplemental Information

Necrostatin-1 Attenuates Renal Ischemia and Reperfusion Injury via Meditation of HIF-1α/mir-26a/TRPC6/PARP1 Signaling

Bingbing Shen, Mei Mei, Youmin Pu, Huhai Zhang, Hong Liu, Maozhi Tang, Qianguang Pan, Yue He, Xiongfei Wu, and Hongwen Zhao

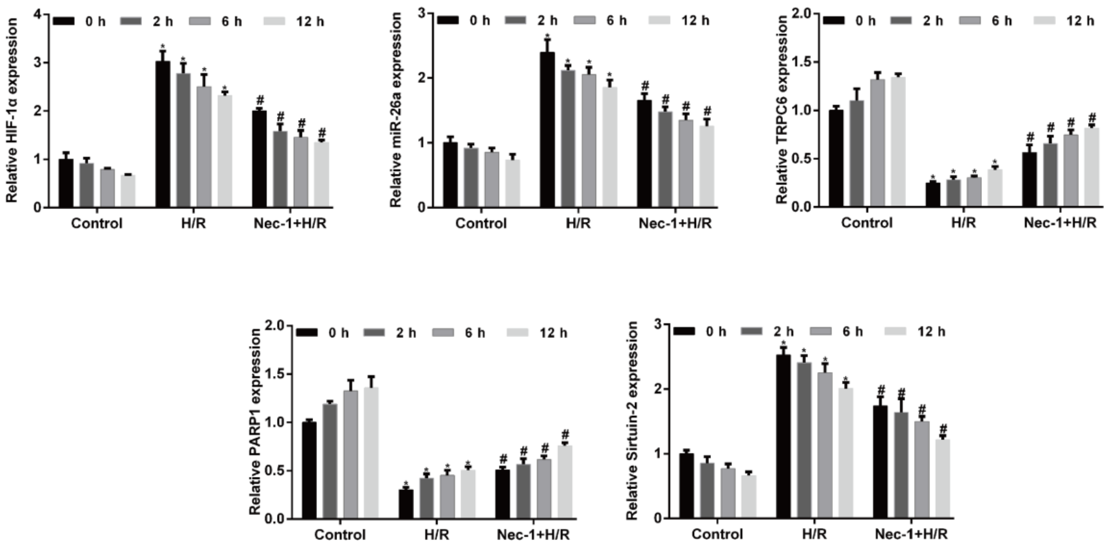

Figure 1S. The levels of HIF-1α, miR-26a, TRPC6, necroptosis, oxidative stress, and inflammation-related molecules in HK-2 cells under conditions of H/R injury with or without Nec-1 treatment. Quantitative real-time PCR was used to determine the relative levels of HIF-1α, miR-26a, TRPC6, PARP1, and Sirtuin-2 in HK-2 cells. *$p < 0.05$ *vs.* control group; #$p < 0.05$ *vs.* the H/R injury group.

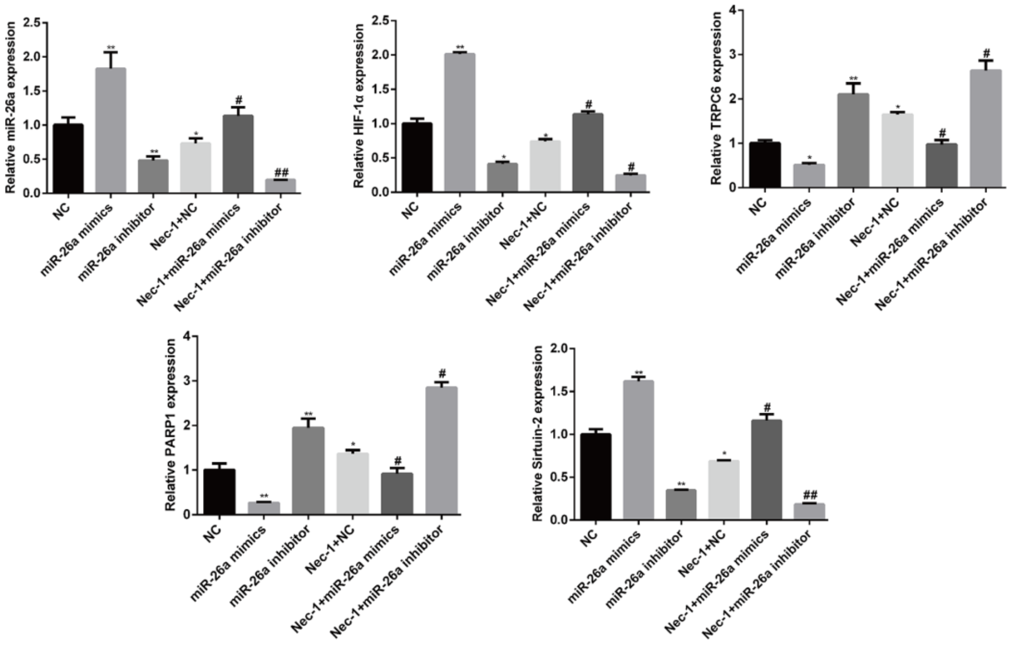

Figure 2S. Effects of downregulation or upregulation of miR-26a on the levels of HIF-1α, miR-26a, TRPC6, necroptosis, oxidative stress, and inflammation-related molecules in HK-2 cells under conditions of H/R injury with or without Nec-1 treatment. Quantitative real-time PCR was used to determine the relative levels of miR-26a, HIF-1α, TRPC6, PARP1, and Sirtuin-2 in HK-2 cells. *$p < 0.05$, **$p < 0.01$ *vs.* the NC group; #$p < 0.05$, ##$p < 0.01$ *vs.* the Nec-1 + H/R group.

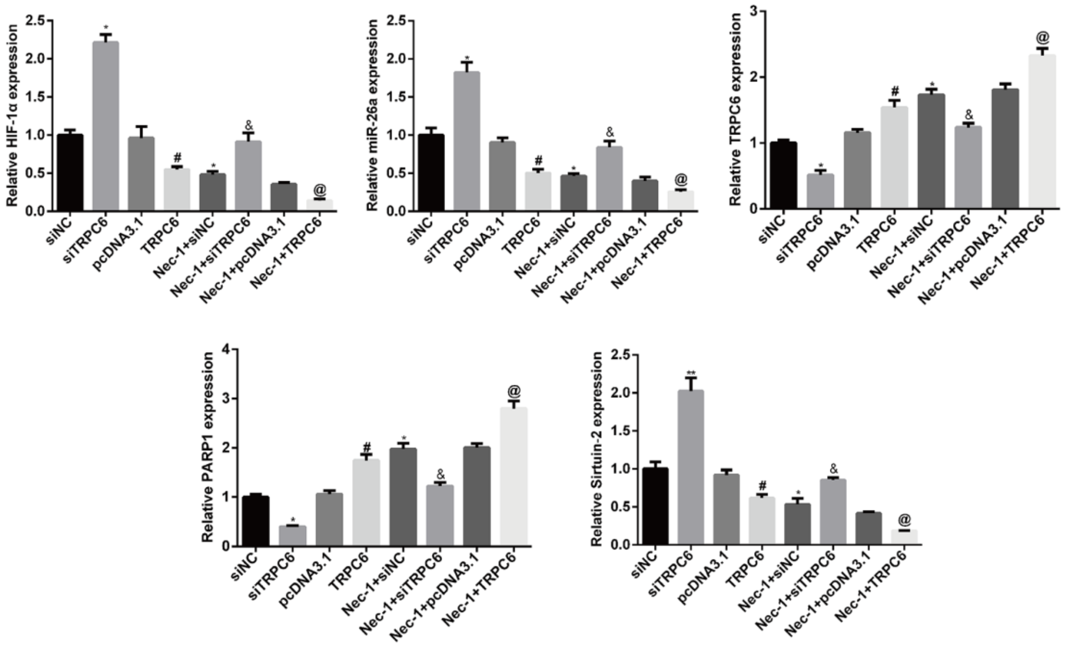

Figure 3S. Effects of TRPC6 overexpression or knockdown on the levels of HIF-1α, miR-26a, TRPC6, necroptosis, oxidative stress and inflammation-related molecules in HK-2 cells under conditions of H/R injury with or without Nec-1 treatment. Quantitative real-time PCR was used to determine the relative levels of miR-26a, HIF-1α, TRPC6, PARP1, and Sirtuin-2 in HK-2 cells. *$p < 0.05$ *vs.* the siNC group; #$p < 0.05$, *vs.* the pcDNA3.1 group; $^{\&}p < 0.05$ *vs.* the Nec-1 + siTRPC6; $^{@}p < 0.05$ *vs.* the Nec-1 + pcDNA3.1 group.

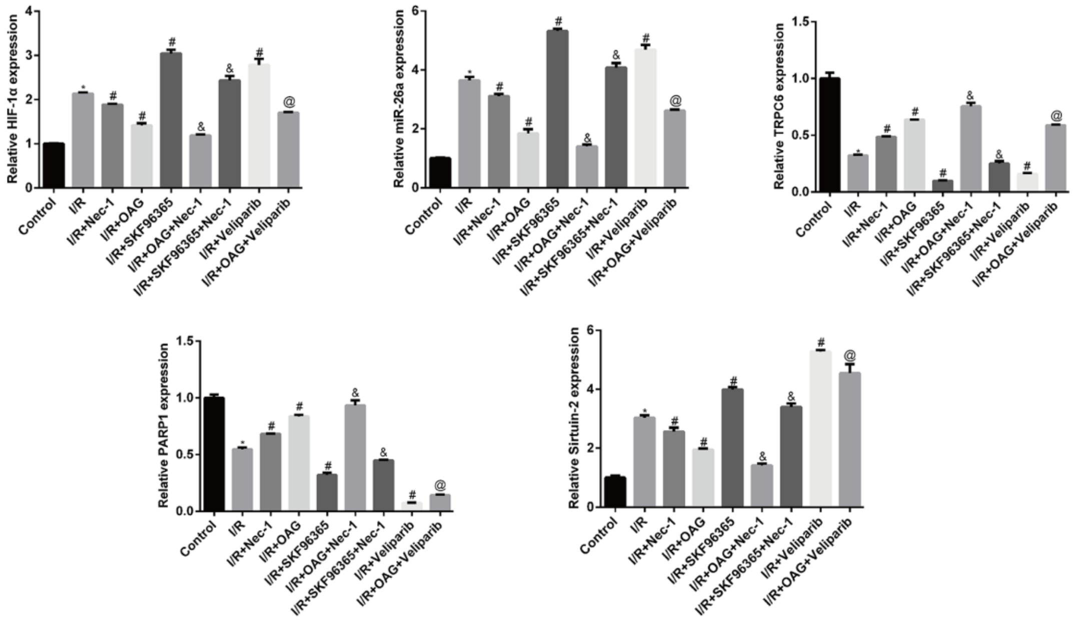

Figure 4S. Nec-1 pretreatment protected rat kidney tissue against I/R-induced oxidative stress and inflammation via the HIF-1α/miR-26a/TRPC6 pathway. Rats in the I/R group were divided into five sub-groups according whether OAG (Sigma, USA), SKF96365 (Sigma, USA) or veliparib (Selleck) was administered before the I/R procedure at 6 h. Similarly, rats in the I/R + Nec-1 group were also divided into three sub-groups. Quantitative real-time PCR was used to determine the relative levels of miR-26a, HIF-1α, TRPC6, PARP1, and Sirtuin-2 in renal tissues. *$p < 0.05$ *vs.* the control group; #$p < 0.05$ *vs.* the I/R group; $^{\&}p < 0.05$ *vs.* the I/R + Nec-1; $^{@}p < 0.05$ *vs.* the I/R + OAG group.

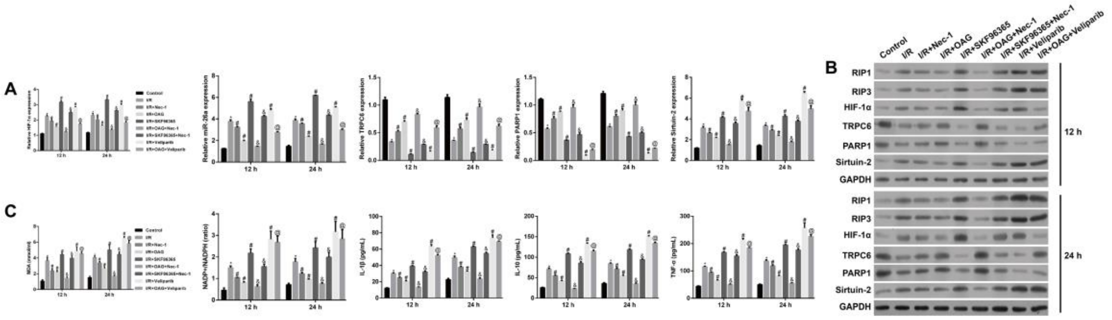

Figure 5S. Nec-1 pretreatment protected rat kidney tissue against I/R-induced oxidative stress and inflammation via the HIF-1α/miR-26a/TRPC6 pathway. Rats in I/R group were divided into five sub-groups according whether OAG (Sigma, USA), SKF96365 (Sigma, USA) or veliparib (Selleck) was administered at before the I/R procedure at 12 and 24 h. Similarly, rats in I/R + Nec-1 group were also divided into three sub-groups. (A) Quantitative real-time PCR was used to determine the relative levels of miR-26a, HIF-1α, TRPC6, PARP1, and Sirtuin-2 in renal tissues. (B) Western blotting was performed to detect HIF-1α, RIP1, RIP3, TRPC6, PARP1, and Sirtuin-2 expression in renal tissues. (C) A MDA detection kit and $NADP^+$/NADPH assay kit were used to measure the MDA level and $NADP^+$/NADPH ratio, respectively. Specific ELISA kits were used to detect the levels of inflammatory cytokines, IL-1β, IL-10, and TNF-α. *$p < 0.05$ *vs.* the control group; #$p < 0.05$ *vs.* the I/R group; $^{\&}p < 0.05$ *vs.* the I/R + Nec-1; $^{@}p < 0.05$ *vs.* the I/R + OAG group.
